# The Relationship between the Measured Blood Concentration of Rocuronium in Stable Muscle Relaxation with a Closed-Loop Control and the Estimated Blood Concentration from a Pharmacokinetic Simulation

**DOI:** 10.3390/jcm13113139

**Published:** 2024-05-27

**Authors:** Yuko Nakanishi, Yuka Matsuki, Osamu Nagata, Shuko Matsuda, Kenji Shigemi

**Affiliations:** 1 Department of Anesthesiology and Reanimatology, Faculty of Medicine Sciences, University of Fukui, Fukui 910-1193, Japan; honjoh@g.u-fukui.ac.jp (Y.N.);; 2 Department of Anesthesia, Touto Kasukabe Hospital, Saitama 344-0022, Japan

**Keywords:** rocuronium, predicted blood concentration, measured blood concentration

## Abstract

We developed a system to adjust the rate of a continuous rocuronium (Rb) infusion to achieve 3 ≤ %T1 ≤ 10 with a closed-loop control. Samples were collected from 15 patients, and Rb blood concentrations were measured at the following time points: (1) when %T1 recovered to 3% or more after the initial Rb infusion; (2) when %T1 stabilized within the target range; (3) at the cessation of the Rb infusion; (4) 5 min after the sugammadex administration. The predicted Rb blood concentration at each time point was calculated and recorded using the pharmacokinetic parameters of Wierda et al. At time points (1), (2), and (3), the predicted blood concentrations were in good agreement with the measured values, but after the administration of sugammadex, the blood concentrations were higher than the predicted values because the Rb distributed in the tissues migrated into the blood. From the above, it was confirmed that the predicted blood concentration of Rb can be a good indicator for the automatic Rb administration control.

## 1. Introduction

Rocuronium bromide (Rb) is not metabolized in the body and is pharmacologically active without producing any by-products, so a stable effect can be obtained with continuous administration. At the same time, large individual differences in the infusion rate are needed to maintain a proper state of muscle relaxation [1]. The pharmacokinetics of Rb are known to follow a three-compartment model [2], and it is possible to calculate the predicted blood concentration from a pharmacokinetic simulation. One method that is effective in maintaining a stable muscle relaxation state that conforms to the differences between individuals is to change the continuous infusion rate as necessary with reference to neuromuscular monitoring and the predicted blood concentration.

We therefore developed an automated infusion adjustment system for Rb that uses pharmacokinetics to maintain a stable muscle relaxation state [3,4]. In this system, the target blood concentration to achieve the optimal state of muscle relaxation is calculated with an information processing application from the predicted blood concentration calculated from a pharmacokinetic simulation based on the constant of Wierda et al. [2] and the information from a neuromuscular monitor. The Rb infusion rate was then automatically adjusted. This allowed the muscle relaxation state to be finely regulated by machine rather than arbitrarily regulated by an anesthesiologist.

In this study, the relationship between the measured and predicted blood concentrations of Rb with an automated infusion adjustment system was investigated under four conditions. Under conditions of stable muscle relaxation, we verified whether the predicted blood concentrations of Rb (pRbs) from a pharmacokinetic simulation and the measured blood concentrations of Rb (mRbs) were in good agreement.

## 2. Materials and Methods

### 2.1. Ethics Approval

This study was approved (20180115) by the ethics committee at the authors’ hospital and was conducted after obtaining written, informed consent from the patients.

### 2.2. Patients

Patients ≥ 20 years old with an American Society of Anesthesiologists (ASA) physical status 1–2 who underwent elective surgery under total intravenous anesthesia at the authors’ hospital between March and June of 2019 were included in this study. The exclusion criteria were as follows: serum electrolyte (Na^+^, Cl^−^, K^+^) abnormalities; possible pregnancy; renal dysfunction (serum creatinine ≥ 2.0 mg·dL^−1^); hepatic dysfunction (AST and ALT greater than two times the upper limit of the hospital’s standard value); emaciation (a BMI ≤ 16 kg·m^−2^); obesity (a body mass index [BMI] ≥ 30 kg·m^−2^); neuromuscular disease; a history of hypersensitivity to rocuronium or bromide; the use of drugs that show interactions with rocuronium; a history of systemic allergy symptoms; and hypothermic anesthesia.

### 2.3. Anesthesia Procedures

A remifentanil infusion was started at 0.5 μg·kg^−1^·min^−1^ after pre-oxygenation using a face mask. Five minutes after the initial remifentanil injection, 0.5 mg·kg^−1^ of propofol was administered for 10 s. The same dose was then administered repeatedly until sleep onset was obtained. Continuous administration of propofol at 10 mg·kg^−1^·h^−1^ was started after sleep was induced. After confirming the loss of consciousness, the ulnar nerve was stimulated at 1 Hz using a neuromuscular monitor (TOF Watch SX; MSD, Tokyo, Japan), and measurements of the T1 twitch height of the adductor pollicis muscle contraction were started. A hand adaptor was attached to the palm and thumb to obtain reproducible measurements. Tetanic stimulation (5 s, 50 Hz) was applied, and the twitch was calibrated to 100% and expressed as a percentage of control after stabilizing the twitch height for the 1-Hz twitch of repetitive stimulation. After the calibration was completed, a rapid intravenous injection of 0.6 mg·kg^−1^ of Rb was performed based on the ideal body weight (IBW) obtained using a BMI of 22 kg·m^−2^. When T1 did not reach 0 within 1 min after the initial administration, an additional 0.3 mg·kg^−1^ of Rb was administered (the total dose of Rb during the induction of the anesthesia was taken to be up to 0.9 mg·kg^−1^). After T1 reached 0, the intubation was performed. Respiration was taken to be controlled after this, and the stimulation frequency of the TOF Watch^®^ SX was changed to 0.1 Hz. A catheter for the blood collection and the direct measurement of the arterial pressure was placed in the radial artery.

### 2.4. Automated Adjustment System

The target T1 was defined to be 3% to 10%. The following procedures were used during the Rb infusion with the automated adjustment system. The rocuronium effect site concentration at the time when T1 recovered to 3% was set as the maintenance target concentration, and continuous infusion of Rb was started so that the predicted rocuronium blood concentration would be at the maintenance target concentration. The continuous infusion rate for Rb was changed together with reassessment of the maintenance target concentration every 12 s, so that T1 would not deviate from the target range (3% ≤ T1 ≤ 10%). In this system, the rocuronium effect site concentration at the time when T1 appeared after the initial administration of rocuronium (TOFC = 1) was set as the initial target value. Then, the target concentration was revised so that T1 = 5. The rocuronium infusion rate was calculated moment by moment with the same algorithm as the target-controlled infusion so that these target concentrations were obtained. When %T1 remained at 0% for 1 min, the Rb target concentration was reduced by 0.05 µg·mL^−1^; when %T1 remained at ≥5% for 1 min, it was increased by 0.1 µg·mL^−1^; and when %T1 remained at ≥8% for 1 min, it was increased by 0.2 µg·mL^−1^. After changing the infusion rate, the target concentration was not changed for 2 min. The continuous infusion rate was then changed according to this algorithm until the end of the surgery. Intraoperatively, the continuous infusion rates of propofol and remifentanil were adjusted to maintain the bispectral index (BIS) between 35 and 55. A forced-air warming system was used to keep the patients’ deep body temperature at ≥35 °C and the skin temperature of the hand on which the acceleration sensor was attached at ≥32 °C. After the surgery was completed, sugammadex at 2 mg·kg^−1^ was administered with T1 at 3–10% together with the termination of the continuous Rb infusion, and the time until the TOF ratio recovered to ≥90% was measured. In cases when the TOF ratio did not reach 90% at 3 min after the administration of sugammadex, an additional 0.5 mg·kg^−1^ of sugammadex was administered. After the TOF ratio recovered to ≥90%, the patient was awakened and extubated.

### 2.5. Blood Concentration Measurements

The blood concentration of Rb was measured: (1) when %T1 recovered to ≥3% after the initial administration of Rb; (2) during the stable period (when T1 was maintained at 3–10% for at least 15 min more than 1 h after the start of the continuous administration of Rb); (3) at the cessation of the Rb infusion; (4) 5 min after the administration of sugammadex.

**Figure 1:**
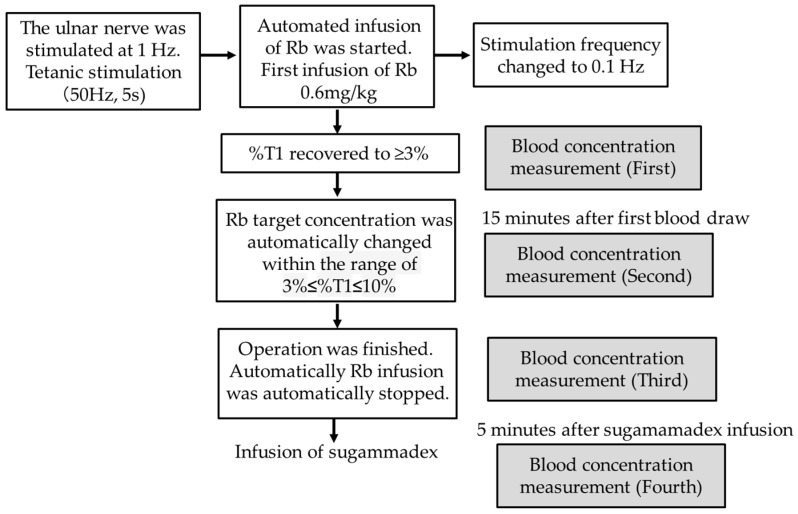
Flow diagram of Rb infusion.

At each time point, 5-mL blood samples were collected from the arterial catheter and stored for the blood concentration measurements. The pRbs at these same times were calculated from pharmacokinetic simulations based on the constant of Wierda et al. [2] and recorded. In this study, a pharmacokinetic simulation was performed for each patient with an automated infusion adjustment system based on a three-compartment pharmacokinetic model, and the predicted blood concentration and effect site concentration were calculated using numerical integration at each time point in accordance with the rocuronium administration history. The rocuronium pharmacokinetic parameters described in [2] were used in this pharmacokinetic simulation. The blood collected to measure the blood concentration of Rb was centrifuged at 3000 rpm for 15 min. Two milliliters of plasma were then extracted, and 0.1 M of sodium dihydrogen phosphate dihydrate (Wako Pure Chemical Industries, Osaka, Japan) was added, after which the specimen was immediately frozen at −20 °C. Rocuronium is a relatively stable substance that does not metabolize or degenerate in the blood, and so there should be no change in concentration even when measured offsite. These specimens were sent to CMIC Pharma Science Co., Ltd. (Yamanashi, Japan), where the blood concentration of Rb was measured using liquid chromatography–tandem mass spectrometry (Figure 1).

### 2.6. Outcome

The relationship between the predicted blood concentrations of Rb at the times when the samples were collected and the measured Rb concentrations in the samples was compared at four time points with a continuous infusion of Rb using the automated infusion adjustment with pharmacokinetics as an indicator.

### 2.7. Sample Size

To estimate the required sample size, a correlation coefficient of about R = 0.475 was assumed based on the results of a study by Kajiura et al. [5], and the type II error rate was set at 20%. The estimated number of cases needed to be 33.

### 2.8. Statistical Analysis

A linear regression analysis was used to investigate the relationship between the measured and predicted blood concentrations under four conditions. The median performance error (MDPE) and median absolute performance error (MDAPE) were used to evaluate the prediction accuracy. The numerical values are shown as mean ± standard deviation values, and *p* values ≤ 5% were considered to indicate significance.

## 3. Results

Although the number of cases needed was first calculated to be 33, the risk of this being excessive was thought to be high, both in terms of the cost and patient-related ethical considerations, and so the analysis was conducted when about half of that number (15 patients) was reached.

Thus, 15 patients were analyzed. The characteristics of the 15 patients are shown in Table 1. There were cases in which blood could not be collected or the timing of the blood collection was inappropriate, so the analysis included (A) 14 patients after the initial infusion, (B) 13 patients during the stable period, (C) 11 patients at the cessation of the Rb infusion, and (D) 11 patients after the administration of sugammadex.

**Figure 2:**
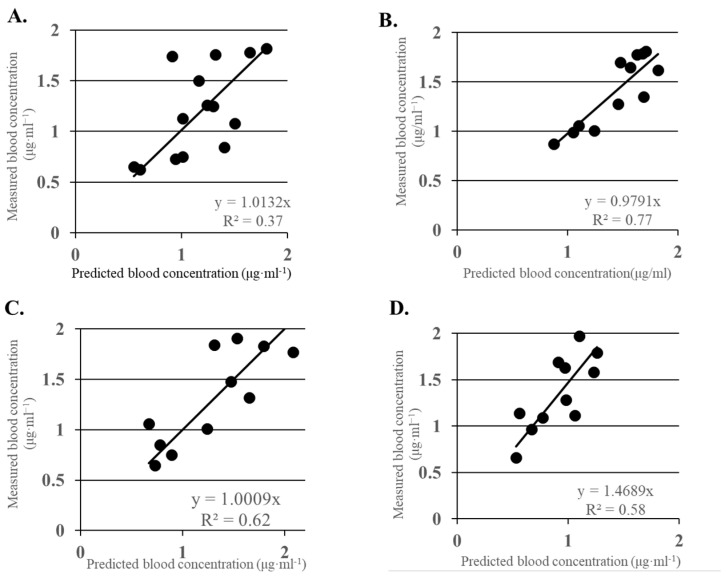
(**A**) Relationship between the predicted and measured Rb blood concentrations at the time %T1 recovered to 3% after the first Rb injection. The mean Rb measured blood concentration is 1.2 ± 0.4 μg·mL^−1^ (95% CI: 0.24–2.18, n = 14). The predicted Rb concentration is proportional to the measured concentration and correlates well (y = 1.01x, r = 0.61, and *p* < 0.05). (**B**) Relationship between the predicted and measured blood concentrations of Rb during the stable period. The mean measured blood concentration is 1.4 ± 0.3 μg·mL^−1^ (95% CI: 0.63–2.19, n = 12). The predicted Rb concentration is proportional to the measured concentration and correlates well (y = 0.98x, r = 0.88, and *p* < 0.05). The MDPE and MDAPE are 2.1% and 19.3%, respectively. (**C**) Relationship between the measured and predicted blood concentrations at the cessation of Rb infusion. The mean Rb measured blood concentration is 1.32±0.5 μg·mL^−1^ (95% CI: 0.25–2.38, n = 11). The predicted concentration is proportional to the measured concentration and correlates well (y = x, r = 0.79, and *p* < 0.05). (**D**) Relationship between the predicted and measured Rb blood concentrations after the infusion of sugammadex. The mean Rb measured blood concentration is 1.36 ± 0.4 μg·mL^−1^ (95% CI: 0.46–2.25, n = 11). A significant linear relationship is seen between the measured and predicted blood concentrations of Rb, though the measured value tends to be higher than the predicted value (y = 1.47x, r = 0.76, and *p* < 0.05).

In four patients, sufficient muscle relaxation during the induction of the anesthesia was not obtained with the initial infusion of Rb and additional infusion was required. The average time until T1 recovered to 3% after it had become 0 with the infusion of Rb was 40 ± 10 min. Figure 2A shows the relationship between the measured and predicted blood concentrations of Rb at the point when %T1 recovered to ≥3% after the initial Rb infusion. The mean predicted and measured blood concentrations were 1.17 ± 0.4 μg·mL^−1^ (95% confidence interval [CI]: 0.4–2.0, n = 14) and 1.2 ± 0.4 μg·mL^−1^ (95% CI: 0.24–2.18, n = 14), respectively. The measured blood concentration and predicted blood concentration correlated well, and the correlation was significant (y = 1.01x, r = 0.61, and *p* < 0.05). The MDPE and MDAPE are indicators showing the discrepancy between the predicted and measured values, and they were 2.1% and 19.3%, respectively.

Figure 2B shows the relationship between the predicted and measured Rb blood concentrations in a state of stable muscle relaxation. The mean predicted and measured blood concentrations were 1.44±0.3 μg·mL^−1^ (95% CI: 0.78–2.11, n = 12) and 1.41 ± 0.4 μg·mL^−1^ (95% CI: 0.63–2.19, n = 12), respectively. The measured and predicted blood concentrations showed a strong correlation (y = 0.98x, r = 0.88, and *p* < 0.05). The MDPE and MDAPE were −2.0% and 7.3%, respectively.

Figure 2C shows the relationship between the predicted and measured blood concentrations at the cessation of the Rb infusion. The mean measured blood concentration was 1.32±0.5 μg·mL^−1^ (95% CI: 0.25–2.38, n = 11). A significant correlation was seen between the measured and predicted blood concentrations (y = x, r = 0.79, and *p* < 0.05), and the MDPE and MDAPE were 1.4% and 18%, respectively.

The mean time until the TOF ratio recovered to ≥90% after the administration of sugammadex was 103 ± 25 s, and, in all patients, the TOF ratio recovered to ≥90% within 3 min. Figure 2D shows the relationship between the measured and predicted values 5 min after the administration of sugammadex. The mean Rb measured blood concentration was 1.36 ± 0.4 μg·mL^−1^ (95% CI: 0.46–2.25, n = 11). A significant correlation was seen between the predicted and measured Rb blood concentrations, though the measured value was significantly higher than the predicted value (y = 1.47x, r = 0.76, and *p* < 0.05). The MDPE and MDAPE were both 29.5%.

## 4. Discussion

This system clarifies the relationship/correlation between the effect site concentration of Rb and the state of muscle relaxation, using a pharmacodynamic model for each individual. This model is derived from the average Rb dosing history across individuals and is used to adjust the dosing accordingly. In this study, a linear relationship was seen between the measured and predicted blood concentrations of Rb during the continuous infusion of Rb with an automated infusion adjustment system using pharmacokinetic analysis. There was general agreement between the two.

Almost no rocuronium is metabolized in the body, and it is excreted unchanged; very little of its metabolite 17-OH is detected [1,2,6]. Thus, the effect of its metabolites can be ignored, meaning the effects of rocuronium can be explained based on pharmacokinetic simulations. In the present study, a pharmacokinetic simulation was conducted using the parameters of Wierda et al. In past studies as well, it was shown that the actual drug effects agreed well with the drug effects in pharmacokinetic simulations using these same parameters [7]. The results for the MDPE and MDAPE in this present study showed that the automated infusion adjustment system using a pharmacokinetic simulation with the parameters of Wierda et al. has a high predictive accuracy and is effective for the automated infusion of Rb.

Blood samples were taken at four time points in this study: (1) when %T1 recovered to ≥3% after the initial administration of Rb; (2) during the stable period (when T1 was maintained at 3–10% for at least 15 min more than 1 h after the start of the continuous administration of Rb); (3) at the cessation of the Rb infusion; (4) 5 min after the administration of sugammadex. The rationale for each of these settings was as follows. For (1), the parameters for pharmacokinetic models are generally obtained from blood concentration measurements when there is a single infusion. Because of that, it was thought that the error between the predicted and measured blood concentrations would be small at the time when %T1 recovered after the initial infusion (the point when %T1 returned to 3%). For (2), it was expected that, in cases when a drug is continuously infused, the measured blood concentration would generally be higher than the mean predicted blood concentration until the drug concentration in the blood from administration becomes evenly distributed. For (3), it was organized in order that one could verify that the predicted blood concentration when the infusion was stably controlled agreed well with the measured blood concentration immediately before the administration of SDX. Finally, for (4), after the antagonist SDX had been administered, the concentration of free Rb in the blood (that is, within the central compartment) decreased because it is encapsulated by the SDX and the Rb in the tissue moves rapidly to the central compartment, where some of that Rb is again encapsulated in the SDX. Thus, the measured blood concentration becomes the total of free Rb and encapsulated Rb. Therefore, it was expected to be higher than the predicted Rb blood concentration calculated in the pharmacokinetic simulation.

The distribution volume is thought to be an important parameter for use in calculating the predicted blood concentration. The concentration in the central compartment that corresponds to the distribution volume is assumed to be uniform, but, in fact, drugs administered intravenously need time for the distribution to become uniform within the compartment. When the time from the infusion of Rb until the collection of blood is very short, the dilution is insufficient, and thus the measured blood concentration is expected to be higher than the predicted blood concentration [8]. The results for the MDPE and the MDAPE at the time when %T1 recovered to ≥3% after the initial infusion of Rb showed that the simulation had a high predictive accuracy, but one of the reasons for this is thought to be that enough time had passed since the initial infusion so that the distribution had become uniform.

It was decided that additional Rb (0.3 mg·kg^−1^) would be administered when %T1 did not reach 0 at 1 min after the initial administration of Rb. The additional dose was administered within a relatively short time. For the four patients in whom Rb was not distributed uniformly due to individual differences in cardiac output, it is thought that sufficient muscle relaxation was not obtained one minute after the initial administration of Rb. In the future, we would like to investigate individual differences in cardiac output using a dynamic cardiac output monitoring system.

In this present study, a significant linear relation was seen between the pRb and mRb, indicating that the Rb automated infusion adjustment system operated properly to adjust the Rb infusion rate. In a previous study, in which the continuous infusion rate of Rb was manually adjusted to achieve an equal muscle relaxation state (3% ≤ T1 ≤ 10%) [5], the mean values of pRb and mRb were 1.7 ± 0.6 μg·mL^−1^ and 1.4 ± 0.4 μg·mL^−1^, respectively. In this present study, the mean values of pRb and mRb were 1.4 ± 0.3 μg·mL^−1^ and 1.4 ± 0.4 μg·mL^−1^, respectively, and the discrepancy between the pRb and mRb was smaller than with the manual adjustment of Rb infusion. This may be because, with the Rb automated infusion adjustment system, the Rb blood concentration could reach the target concentration in a short time and the target concentration could be stably maintained, because the target concentration changed frequently and automatically.

The differences between the measured and predicted blood concentrations also result from relative changes in the distribution volume. They are affected not only by the different conditions of each patient, such as age, sex, and body fat percentage (the differences between individuals), but also by factors that change even within the same patient, such as the body temperature, heart rate, and circulating blood volume (the differences within the same individuals). In past studies as well, it was reported that increases and decreases in the cardiac stroke volume affects blood concentrations during the continuous infusion of anesthesia [9,10]. Even if the drug is distributed within a minute, each minute of continuous drug administration is considered to be affected as long as the drug is administered continuously. Meanwhile, the measured values would be expected to be higher than the predicted values during the continuous infusion of a drug, since dilution continues to be insufficient. Given that there was good agreement between the measured and predicted values in the stable period and at the cessation of the Rb infusion, the effect of inadequate dilution was minor.

The mean effective site concentration of Rb was 1.2 ± 0.4 μg·mL^−1^ at the time %T1 recovered to 3% after the first dose, with values ranging from 0.6 to 2.2 μg·mL^−1^, indicating an individual difference of approximately 3.7-fold in this study. Similar to our findings, a previous study demonstrated that a 3-fold difference in the Rb infusion rate is required to maintain %T1 at 3–10% [5]. Considering these individual differences, this present study used an automated Rb infusion system capable of individual Rb infusions to monitor the degree of muscle relaxation and to maintain %T1 between 3 and 10%.

In measuring the blood concentration of Rb, the assay does not differentiate between sugammadex-encapsulated Rb and unencapsulated (free) Rb; both forms are measured collectively as the total Rb. When sugammadex is encapsulated with Rb, the blood concentration of free Rb decreases and the Rb at neural junctions moves into the blood according to the concentration gradient [11]. One reason explaining why the measured blood concentration of Rb is higher than the predicted blood concentration after the administration of sugammadex is thought to be that Rb moves from the tissue into the blood with sugammadex encapsulation, and the total of the encapsulated Rb and free Rb greatly surpasses the predicted value.

There are some limitations to this study. First, with the automated adjustment of infusion, the target concentration and infusion rate change moment to moment, and thus the infusion might sometimes have a high flow rate and might sometimes be stopped. In cases such as when there is a short time from the infusion at a high flow rate until the blood is collected, the drug may not be distributed uniformly and there is thought to be a risk that the measured blood concentration will be higher than the predicted blood concentration. When comparing the blood concentrations during the continuous infusion with manual adjustment, measures such as collecting blood after a certain time has passed from a change in the infusion rate were taken. However, with an automated infusion adjustment system, the infusion rate changes with timing that is not anticipated by the anesthesiologist, so in the stable period and before the administration of sugammadex, this kind of measure regarding the timing of blood collection cannot be taken. Second, the pharmacokinetic simulation used in this present study did not include the effects of sugammadex encapsulation. In the future, we would like to add data and prepare simulations that include an antagonist using the analytical models of Kleijn and Wierda and conduct investigations that also include the calculation of the blood concentration of free Rb. Third, the anesthesia in this study was total intravenous anesthesia. Inhalational anesthetics have a muscle relaxation effect, suggesting the possibility that the same results would not be obtained if inhalational anesthesia were used.

## 5. Conclusions

If the muscle relaxation state is controlled using the Rb effect site concentration as an index, which is calculated using the pharmacokinetic parameters obtained from a population analysis, then the muscle relaxation state can be appropriately controlled. In addition, the predicted blood concentration of Rb also correlates well with the actual measured blood concentrations of Rb. Consequently, we propose that this automated infusion system has a practical utility in clinical settings.

## Figures and Tables

**Table 1:** Patients’ characteristics.

Characteristic (*n* = 15)	
Sex (Female/Male)	10/5
Age (y)	59 ± 15 (35–81)
Weight (kg)	63 ± 10 (45–78)
Height (cm)	161 ± 8 (150–175)
BMI (kg·m^−2^)	24 ± 3 (20–27)
Rocuronium duration time (minutes)	146 ± 82 (72–367)
Rocuronium total dose (mg)	101 ± 33 (61–169)
Rocuronium continuous dosing rate (µg·kg^−1^·min^−1^)	7.5 ± 3.7 (3.7–18.5)

BMI: body mass index. Mean ± SD (range).

## Data Availability

The datasets used and/or analyzed during this current study are available from the corresponding author on reasonable request.

## References

[1] McCoy E.P., Mirakhur R.K., Maddineni V.R., Wierda J.M., Proost J.H. (1996). Pharmacokinetics of rocuronium after bolus and continuous infusion during halothane anaesthesia. Br. J. Anaesth..

[2] Wierda J.M.K.H., Kleef U.W., Lambalk L.M., Kloppenburg W.D., Agoston S. (1991). The pharmacodynamics and pharmacokinetics of Org 9426, a new non-depolarizing neuromuscular blocking agent, in patients anaesthetized with nitrous oxide, halothane and fentanyl. Can. J. Anaesth..

[3] Matsuki Y., Nagata O., Ogino Y., Shigemi K. (2021). Development of an automated rocuronium infusion system and evaluation of its accuracy. J. Clin. Anesth..

[4] Nagata O., Matsuki Y., Ogino Y., Shigemi K. (2022). Safety and efficacy of an automated anesthesia delivery system for total intravenous anesthesia with propofol, remifentanil, and rocuronium: A non-inferiority randomized controlled trial versus manually controlled anesthesia. J. Anesth..

[5] Kajiura A., Nagata O., Takizawa Y., Nakatomi T., Kodera S., Nakayama T. (2015). A large individual variation in both the infusion rate and the blood concentration of rocuronium necessary for obtain adequate surgical muscle relaxation during total intravenous anesthesia with propofol and remifentanil. J. Anesth..

[6] Proost J., Eriksson L., Mirakhur R., Roest G., Wierda J. (2000). Urinary, biliary and faecal excretion of rocuronium in humans. Br. J. Anaesth..

[7] Vermeyen K.M., Hoffman V.L., Salfien V. (2003). Target controlled infusion of rocuronium: Analysis of effect data to select a pharmacokinetic model. Br. J. Anaesth..

[8] Kuipers J.A., Boer F., Olofsen E., Bovill J.G., Burm A.G.L. (2001). Recirculatory pharmacokinetics and pharmacodynamics of rocuronium in patients: The influence of cardiac output. Anesthesiology.

[9] Kurita T., Morita K., Kazama T., Sato S. (2002). Influence of cardiac output on plasma propofol concentrations during constant infusion in swine. Anesthesiology.

[10] Birkholz T., Leuthold C., Schmidt J., Ihmsen H., Schüttler J., Jeleazcov C. (2018). Influence of cardiac output on the pharmacokinetics of sufentanil in anesthetized pigs. Anesthesiology.

[11] Epemolu O., Bom A., Hope F., Mason R. (2003). Reversal of neuromuscular blockade and simultaneous increase in plasma rocuronium concentration after the intravenous infusion of the novel reversal agent Org 25969. Anesthesiology.

